# Bayesian Approach to Assessing Uncertainty and Calculating a Reference Value in Key Comparison Experiments

**DOI:** 10.6028/jres.110.085

**Published:** 2005-12-01

**Authors:** Blaza Toman

**Affiliations:** National Institute of Standards and Technology, Gaithersburg, MD 20899-8980

**Keywords:** Bayesian hierarchical models, linear opinion pool, Markov Chain Monte Carlo methods, synthesis of probability distributions

## Abstract

International experiments called Key Comparisons pose an interesting statistical problem, the estimation of a quantity called a Reference Value. There are many possible forms that this estimator can take. Recently, this topic has received much international attention. In this paper, it is argued that a fully Bayesian approach to this problem is compatible with the current practice of metrology, and can easily be used to create statistical models which satisfy the varied properties and assumptions of these experiments.

## 1. Introduction

In October of 1999, the directors of the national metrology institutes of 38 of the member states of the *Metre Convention*, signed a document called the *Mutual Recognition Agreement* (MRA) [1] dealing with national measurement standards. The objectives of the MRA are the establishment of degree of equivalence of national measurement standards maintained by the national metrology institutes, the recognition of calibration and measurement services provided by the institutes and consequently, the establishment of a secure technical foundation for wider agreements related to international trade. The process of achieving these objectives is through international comparisons of measurements called Key Comparisons. The overall coordination of the Key Comparisons is through the International Bureau for Weights and Measures (BIPM) under the authority of the International Committee for Weights and Measures (CIPM). Details of the agreements can be found on the BIPM website, http://www.bipm.fr.

There are general guidelines, given in Ref. [2], for the execution of the Key Comparison experiments, dealing with the selection of what is to be measured, who is to participate and how the results are to be disseminated. There are now Key Comparison experiments being performed in a great variety of disciplines. Some examples are temperature measurements, gas flow measurements, responsivity of photodiodes to various wavelengths of light, sound measurements and many more. Two classes of Key Comparisons have been defined in Refs. [3, 4]. A Class 1 Comparison is one where each participant measures a local standard, or possibly a traveling artifact. In such an experiment there may be a single quantity that is being measured (measurand) if the artifact is stable, but if not, there may be large systematic laboratory effects which need to be accounted for in the statistical model and analysis. Currently, most Key Comparisons belong to Class 1 and an example is given in Sec. 3. A Class 2 Comparison is one where all laboratories take measurements of a single physical state or property. Thus in a Class 2 experiment, there is a clearly defined single measurand.

In either type of comparison, the laboratories perform their measurements and report their results back to a pilot laboratory. The results from each laboratory consist of an average of a set of measurements (often with a small sample size) of the quantity being measured and the total uncertainty in the measurements. The calculation of the *uncertainty* is prescribed in the *Guide to the Expression of Uncertainty in Measurement* [5], a publication of the ISO. The pilot laboratory assembles all of the data and calculates various measures of agreement among the participating laboratories. The exact form of the analysis is not prescribed, and there is considerable debate in the metrology community about this issue. However, it is stated in Ref. [2] that the pilot laboratory writes a report on the key comparison that should include “proposals for a reference value,” called the Key Comparison Reference Value (KCRV). The KCRV is said to be “usually a close approximation to the corresponding SI value.” In a Class 2 Key Comparison, the KCRV is clearly an estimate of the measurand. Class 1 Key Comparisons may have multiple measurands and so the interpretation of the KCRV is not straightforward. To date, there have been numerous proposals for the calculation of the KCRV. The articles given in Refs. [6–10] present some of the methods and more can be found in their references. The published methods have so far been based mostly on frequentist statistical models, even though, the *Guide* uses belief-based definition of probability, and so can be said to be more compatible with Bayesian statistical methods.

This paper proposes fully Bayesian models for Key Comparison data analysis. It is shown that their assumptions are compatible with the approach of the *Guide*. The models allow for a unified approach to the analysis of Class 1 Key Comparisons, whether or not systematic laboratory effects are present. In Sec. 2, the *Guide’s* definition of measurement uncertainty is fully described and a compatible Bayesian mathematical model for measurement is presented. Sec. 3 contains an example of a Key Comparison experiment. Sec. 4 gives the statistical models for Key Comparison data. Sec. 5 presents the analysis for Key Comparisons with a single and with multiple measurands. Sec. 6 presents the analysis of the example Key Comparison. Conclusions follow in Sec. 7.

## 2. The Measurement Model

The approach to quantification of uncertainty in measurement, which is now widely used in the physical sciences, is that presented in the *Guide to the Expression of Uncertainty in Measurement.* The basic idea of the *Guide* is to approximate a measurement equation

$$\mu =g\left(\theta_{1},\ldots ,\theta_{p}\right),$$ (1)

where *g* is a known function, *μ* denotes the measurand and *θ*_1_,…,*θ_p_* denote *p* input quantities (random variables), by a first order Taylor series about the expected values of the *θ_i_* s. The uncertainty in the measurand, denoted *u*(*μ*), is then defined as the standard deviation of the probability distribution of *μ* based on this linear approximation, that is

$$u\left(\mu \right)=\sqrt{\sum c_{i}^{2}\;\mathrm{var}\;\left(\theta_{i}\right)+2\sum_{i<j}c_{i}c_{j}\;\mathrm{cov}\;\left(\theta_{i},\theta_{j}\right)},$$ (2)

where the *c_i_* are the partial derivatives of *μ* with respect to *θ_i_*, var denotes the variance, and cov denotes the covariance. The *Guide* uses an interpretation of probability consistent with the Bayesian paradigm, that is, probability distributions of the *θ_i_* and *μ* summarize our knowledge about these quantities. The expected values and variances of the *θ_i_* may be based on actual physical measurements or on other information such as expert opinion. The *Guide* defines two types of uncertainty evaluations. Type A is “by the statistical analysis of series of observations” and this has usually been interpreted as “using sample standard deviations.” Type B is by “other means” and this has usually meant using manufacturer specifications, expert knowledge or even data from additional experiments. An example of a Type B evaluated uncertainty is the uncertainty in the internal volume of a 100 ml flask being used in a chemistry experiment. Here, the manufacturer may give a volume of 100 ml ± 0.1 ml. This could be interpreted as the volume having a rectangular distribution on the interval (99.9 ml, 100.1 ml), that is, having a standard deviation of 0.058. A key idea is that the data from the present experiment is not informative about sources of uncertainty evaluated by Type B methods. Such uncertainty is due to systematic effects that influence all of the observations in the experiment, such as for example, a flask not really having a volume of 100 ml.

The most common analysis of a metrology experiment estimates the expected value of *μ* by *y*, the output of the measurement equation

$$y=g\left(x_{1},\ldots x_{r},\lambda_{1},\ldots ,\lambda_{s}\right)$$ (3)

where *p* = *r* + s, *r* of the input quantities having physical measurements and *s* of the input quantities being based on other information. The *x_i_* represent sample means of the measurements used to estimate *θ_i_*, *λ_i_* represent the subjective evaluations of the means of the remaining input quantities. The *u*(*μ*) is approximated as *u*(*y*) as follows. The variances of the frequency-based distributions of the *r* sample means are usually used for the corresponding variances in Eq. (2). Subjective evaluations are used for the variances of the remaining *θ_i_*’s. The usual interpretation of the *y* and the *u*(*y*) is as the mean and standard deviation of a probability distribution of the measurand. From a statistical perspective, this usage represents methodology that is neither totally frequentist nor totally Bayesian, but can be viewed as an approximate solution to a Bayesian inference problem. For further discussion of this subject see Refs. [11, 12].

In the analysis of Key Comparison experiments, the pilot laboratory receives the values *y* and the uncertainties *u*(*y*) from all of the participants. Generally, the participants can also provide an estimate of the repeatability component of the uncertainty. Even though, it is understood that *y* and *u*(*y*) are features of a probability distribution for a measurand, no information about the shape of this distribution is provided. For a single laboratory, the following Bayesian statistical model gives results consistent with the *Guide’s* measurement model, and has the added benefit of being easily extended to accomplish the KCRV estimation

$$\begin{array}{l} Y|\theta ,\sigma^{2}\sim N\left(\theta ,\sigma^{2}\right)\\ \theta |\mu ,\tau^{2}\sim N\left(\mu ,\tau^{2}\right)\\ \mu |m,\omega^{2}\sim N\left(m,\omega^{2}\right). \end{array}$$ (4)

The notation *Y* |*θ*,*σ*^2^ ~ *f*(*Y* |*θ*,*σ*^2^) = *N*(*θ*,*σ*^2^) represents conditioning. That is, the probability distribution of *Y*, given *θ* and *σ*^2^, is *f*(*Y*|*θ*,*σ*^2^) which is a Gaussian (Normal) distribution with mean *θ* and variance *σ*^2^. The participant’s inputs are a sample mean *y*, a sample standard deviation *s*^2^ (an estimate of *σ*^2^) and the remaining uncertainty *τ*^2^. In this model, stage one in the hierarchy is used to quantify the usual sampling variability of *y*, that is, the uncertainty component due to repeatability. Stage two represents the remaining uncertainty, both that evaluated by Type A and Type B methods. Stage three is a prior distribution of the measurand *μ*. Normal distributions are used in this article but other forms of probability distributions can easily be substituted when appropriate. Generally, a non-informative stage three prior distribution on *μ* would be used, that is, allow *ω*^2^ → ∞. Application of Bayes Theorem

$$f\left(\mu |y\right)=\frac{f\left(y|\theta ,\sigma^{2}\right)f\left(\theta |\mu ,\tau^{2}\right)f\left(\mu \right)}{\int f\left(y|\theta ,\sigma^{2}\right)f\left(\theta |\mu ,\tau^{2}\right)f\left(\mu \right)d\mu }$$

leads to a posterior distribution for *μ*

$$\mu |y,\tau^{2},\sigma^{2}\sim N\left(y,\tau^{2}+\sigma^{2}\right).$$ (5)

In the above model the variance *σ*^2^ is assumed to be a known quantity. As this is generally not true, *σ*^2^ would be estimated by the sample variance *s*^2^. When *τ*^2^ dominates *σ*^2^, as is often the case in high precision physical measurements, or when the sample size on which *s*^2^ is based is large, the posterior distribution of *μ* can be well approximated by

$$\mu |y,\tau^{2}\sim N\left(y,\tau^{2}+s^{2}\right).$$ (6)

(When the relationship between *τ*^2^ and *σ*^2^ is less extreme, it is better to assign *σ*^2^ a non-informative prior distribution and obtain the posterior distribution of *μ* by Markov Chain Monte Carlo methods. This will be shown in an example).

Thus the approximate posterior mean and posterior standard deviation arising from Eq. (4) are in fact the quantities recommended for estimation by the *Guide*. In the next section, an example of a Key Comparison experiment is described in order to motivate the proposed modeling and analysis.

## 3. Example of a Key Comparison Experiments, Vibration Acceleration (CCAUV.V-K1)

The aim of this experiment in the area of vibration and shock measurement was to compare measurements of sinusoidal linear accelerations in the frequency range from 40 Hz to 5 kHz. During the period from January 2000 to June 2001, 12 national metrology institutes used two different accelerometers, one of single-ended design and one of back-to-back design, to measure charge sensitivity at different frequencies. The charge sensitivity was given in *pico coulomb per meters per second squared* [pC/(m/s^2^)]. All laboratories followed the same measurement protocol, controlling temperature and relative humidity and other external variables which could affect the measurements. The German institute Physikalisch Technische Bundesanstalt (PTB) was the pilot laboratory, responsible for checking the long term stability of the accelerometers. These were hand-carried from the pilot laboratory to the participating institutes in a closed box by representatives of the various institutes. The data from the Key Comparison was published in *Report on Key Comparison CCAUV.V-K1* [13] and is now available for further study. This is a Class 1 Key Comparison with a traveling artifact, which can be considered as having a single measurand.

A subset of this data, charge sensitivity for the single-ended accelerometer at 40 Hz, is given here in Table 1. The table contains the mean values (*y_i_*), the repeatability component of the uncertainty (*s_i_*) and the Type B evaluated uncertainties (*τ_i_*) for this measurement.

The mean measurements are averages over five measurements for all but three laboratories. Laboratory one took nine measurements, laboratory eight took three measurements, and laboratory nine took four measurements. The *s_i_* are the sample standard deviations of the means. Each laboratory calculated their uncertainty values by the usual error propagation techniques, see Ref. [5], and included terms such as uncertainty from possible voltage disturbances, phase noise, uncertainty in the vibration frequency measurement and others. All of the possible sources of uncertainty that were to be considered are described in the publication [14]. Each laboratory assessed their Type B uncertainty independently of the other laboratories and without knowledge of the other laboratories’ measurements.

## 4. Two Models for Key Comparison Data

### 4.1 Multiple Means Model

A Key Comparison experiment is a multi-laboratory study. If we treat all laboratories data totally independently from each other, that is, if we assume that there are no relationships between the measurands or the uncertainties of the various laboratories, we can extend the statistical model given in Eq. (4) as follows. For *i* = 1,…, *k*, where *k* is the number of laboratories, we have

$$\begin{array}{l} Y_{i}|\theta_{i},\sigma_{i}^{2}\sim N\left(\theta_{i},\sigma_{i}^{2}\right)\\ \theta_{i}|\mu_{i},\tau_{i}^{2}\sim N\left(\mu_{i},\tau_{i}^{2}\right)\\ \mu_{i}|m_{i},\omega^{2}\sim N\left(m_{i},\omega^{2}\right) \end{array}$$ (7)

The posterior distributions of the *μ_i_* can be approximated by

$$\mu_{i}|y_{i},\tau_{i}^{2}\sim N\left(y_{i},\tau_{i}^{2}+s_{i}^{2}\right),$$ (8)

and so the standard uncertainty of each laboratory is approximately the *Guide* recommended quantity.

### 4.2 Single Mean Experiment

Suppose now that there is a common measurand as would be true in all Class 2 Key Comparisons and in most Class 1 Key Comparisons. Equation (7) can be modified to reflect this fact:

$$\begin{array}{c} \begin{array}{l} Y_{i}|\theta_{i},\sigma_{i}^{2}\sim N\left(\theta_{i},\sigma_{i}^{2}\right)\\ \theta_{i}|\mu_{i},\tau_{i}^{2}\sim N\left(\mu_{i},\tau_{i}^{2}\right)\\ \mu_{i}|\mu ,\gamma^{2}\sim N\left(\mu ,\gamma^{2}\right) \end{array}\\ \begin{array}{l} \mu \sim N\left(m,\omega^{2}\right)\\ \gamma \sim U\left(0,c\right). \end{array} \end{array}$$ (9)

The notation *U*(0, *c*) represents a rectangular (Uniform) distribution on the interval (0, *c*), where *c* is a constant. The prior distributions on the *μ_i_* are now hierarchical, the common mean *μ* being the measurand of the entire experiment. Note that *γ*^2^ represents variability due to systematic laboratory effects. When a prior distribution for it is specified, *γ*^2^ can be estimated from the combined data of the participating laboratories. Recall that for each individual laboratory, the contribution of such systematic laboratory effects to their uncertainty is not estimable by their own data and so is evaluated by Type B methods and is part of *τ_i_*. For example, in the CCAUV.V-K1 key comparison, the uncertainty in the vibration frequency measurement is of this type. So is essentially any uncertainty attributed to the individual laboratory’s technique. Thus the uncertainty estimate of *μ* based on Eq. (9) is somewhat conservative as it is impossible, without a complete list of all Type B evaluated uncertainties from each participant, to separate out the effects which are estimable from the pooled data and the effects which are not. When the *σ*^2^*_i_* are considered unknown and are given prior distributions, analysis based on Eq. (9) must be done numerically as closed form solutions are not available. Markov Chain Monte Carlo methods can readily be used, see Ref. [15] for sample computer programs.

A special case of Eq. (9) is when *γ*^2^ = 0, that is, when all laboratories are assumed to be truly measuring the same quantity. In such a case, allowing *ω*^2^ → ∞ and following Ref. [16], it can be shown that approximately, the posterior distribution of *μ* is normal with mean *μ_p_* and standard deviation *ω_p_* where

$$\mu_{p}=\frac{\sum_{1}^{k}y_{i}{\left(\tau_{i}^{2}+s_{i}^{2}\right)}^{-1}}{\sum_{i}^{k}{\left(\tau_{i}^{2}+s_{i}^{2}\right)}^{-1}}$$ (10)

$$\omega_{p}=\frac{1}{\sum_{i}^{k}{\left(\tau_{i}^{2}+s_{i}^{2}\right)}^{-1}}.$$ (11)

## 5. Analysis

### 5.1 Single Measurand Experiment

Both Eqs. (7) and (9) can be used to construct a KCRV and its uncertainty for a single measurand experiment.

First, consider using Eq. (9). In this case, the meaning of the KCRV is clear and so is its estimation. It is plainly an estimate of the common measurand *μ* and can be provided by the mean of the posterior distribution. The uncertainty is the posterior standard deviation of *μ*. If *γ*^2^ is set to 0, the solution [Eq.(10)] is the most commonly used KCRV estimate described in Ref. [6]. However, unlike in that publication, here it is derived based on a Bayesian model. The underlying assumption of a common laboratory mean has been questioned in the literature, see for example Ref. [17]. Equation (9) with a prior distribution on *γ*^2^ provides a sensible alternative, one that allows for systematic laboratory differences, provides a more conservative estimate of the uncertainty of the KCRV and also allows for a degree of validation of the stated uncertainties. The publication [17] takes another approach and directly models the differences between the measurand *μ* and the *μ_i_*. The resulting KCRV is somewhat related to that described in Sec. 5.2.

It is possible that the participants of the Key Comparison may not wish to allow the mathematical model to pool their data automatically, but want to determine the form of the KCRV more directly. An alternate approach then would be to use the multiple means Eq. (7) and to construct a KCRV based on it. This requires a synthesis of the probability distributions of the *μ_i_* into a single distribution for *μ*. The literature on such methods is rich and has been reviewed in Refs. [18] and [19]. An approach that is sensible for the current application is the *Supra-Bayesian* technique given in Ref. [20]. This can be described as follows:

A single person with vague prior knowledge of a parameter *μ* consults *k* experts who provide the means (in our notation *y_i_*) and standard deviations (in our notation $\sqrt{\tau_{i}^{2}+s_{i}^{2}}$) of their probability distributions for *μ*. The person then combines the *k* experts’ distributions into a single probability distribution. He does this by first specifying a normal likelihood function to express his opinion about the experts’ knowledge and then using Bayes Theorem. Namely he specifies that the distribution

$$p\left(y_{1},\ldots ,y_{k}|\tau_{1},\ldots \tau_{k},s_{1},\ldots ,s_{k},\mu \right)$$ (12)

is multivariate normal with means *α_i_* + *β_i_μ*, standard deviations $\kappa_{i}\sqrt{\tau_{i}^{2}+s_{i}^{2}}$ and correlations *ρ_ji_* for *i* = 1,…,k. In this way he can express his beliefs about the possible biases (in terms of the *α_i_* and *β_i_*) of the experts, about their precision (in terms of *κ_i_*), and to what extent their assessments are correlated or not. In the case of no correlation between the laboratories, the resulting posterior distribution for *μ* is normal with mean

$$\mu_{B}=\frac{\sum_{1}^{k}\beta_{i}\left(y_{i}-\alpha_{i}\right)\kappa_{i}^{-2}{\left(\tau_{i}^{2}+s_{i}^{2}\right)}^{-1}}{\sum_{1}^{k}\beta_{i}^{2}\kappa_{i}^{-2}{\left(\tau_{i}^{2}+s_{i}^{2}\right)}^{-1}}$$ (13)

and variance

$$\omega_{B}=\frac{1}{\sum_{1}^{k}\beta_{i}^{2}\kappa_{i}^{-2}{\left(\tau_{i}^{2}+s_{i}^{2}\right)}^{-1}}.$$ (14)

Note that when the *α_i_* are set to 0, and the *β_i_* and *κ_i_* are set equal to one, these expressions become Eqs. (10) and (11). Note that this is again the *weighted mean estimator* as described in [6]. The most frequent criticism of this analysis is based on the belief that the values of the Type B uncertainties cannot be considered as well determined quantities but rather only as estimates of the underlying variability. Taking the *κ_i_* not as constants but giving them a probability distribution can model this fact.

For simplicity, it will be assumed here that the laboratories’ results are independent and thus that the *ρ_i_* are equal to 0. This is generally a reasonable assumption in Key Comparisons but can be relaxed if necessary. Reference [20] shows that without loss of generality, the *α_i_* may be set to 0 and the *β_i_* to 1, when the following probability model for the *κ_i_* is employed:

$$\frac{\nu_{i}\;c_{i}^{2}}{\kappa_{i}^{2}}\sim \chi_{\upsilon_{i}}.$$ (15)

Note that $E\left(\kappa_{i}^{2}\right)=c_{i}^{2}\nu_{i}/\left(\nu_{i}-2\right)$ and the coefficient of variation of *κ_i_* is (*ν*/2 − 2)^−1/2^. The values of *c_i_* and the degrees of freedom *ν_i_* therefore specify the location and the spread of the distribution of *κ_i_*. The selection of the values can be aided by noting that *κ_i_* is approximately normal with mean log *c_i_* and variance (2*ν_i_*)^−1^, which further implies that approximately, $a_{i}^{-1}c_{i}<\kappa_{i}<a_{i}c_{i}$, for *a_i_* such that $\log\;a_{i}=\sqrt{2/\nu_{i}}$. Further discussion of these relationships appears in Ref. [20] and also in Ref. [21].

Combining the likelihood function [Eq. (12)] and the prior distribution [Eq. (15)] via Bayes Theorem for a *single laboratory i* results in

(μ−yi)ci(τi2+si2) (16)

having a student *t_υi_* distribution. Because of the independence of the laboratories, using Bayes Theorem with Eqs. (12) and (15) for all *k* laboratories results in a posterior distribution of *μ* which is a product of the *t_υi_* (*i* = *l*,…, *k*) distributions. An interesting property of this distribution is that it can be multi-modal when there is strong disagreement among the laboratories. This model, in a more general context, is also discussed in some detail in Ref. [22] and further generalized by Ref. [23].

The KCRV can be taken to be the mean or possibly the median of this distribution. The uncertainty is the standard deviation of this distribution. Such quantities cannot be obtained in closed form but can easily be computed using standard Markov Chain Monte Carlo methods (Ref. [15]). Interestingly, the same distribution was derived using a different model and different motivation by [24] for the problem of combining data which appear to be in mutual disagreement.

Both approaches, that is KCRV estimation based on Eq. (9) or the Supra-Bayesian KCRV based on Eq. (7), are reasonable. Arguably, the Supra-Bayesian method introduces fewer assumptions, and allows a more direct modeling of possible inaccuracies in the individual Type B evaluations. On the other hand, the straightforward interpretation of Eq. (9) and the possibility of data-based estimation of the effects of the systematic laboratory effects makes this approach appealing as well. Analysis of data from CCAUV.V-K1 key comparison in Sec. 6 will illustrate the two approaches.

### 5.2 Multiple Measurand Experiments

In some Class 1 experiments there are clearly defined multiple measurands. In such a case, Eq. (7) would be used. The question then is again how to estimate the KCRV since it has no natural interpretation. The Supra-Bayesian solution given in Sec. 5.1 is not applicable here since there is no common measurand. One possible solution is the following method, based on the so-called *linear opinion pool*, which dates back to Laplace. In this method *k* probability distributions *p_i_* () are combined as

$$p\left(\;\right)=\sum_{i=1}^{k}w_{i}p_{i}\left(\;\right)$$ (17)

where the weights *w_i_* add up to one. In the present application, the *k* laboratories posterior distributions for *μ_i_* could be combined into the mixture distribution of a new random variable *μ*, namely

$$p\left(\mu \right)=\sum_{i=1}^{k}w_{i}\frac{1}{\sqrt{2\pi \left(\tau_{i}^{2}+s_{i}^{2}\right)}}e^{-\frac{{\left(\mu -y_{i}\right)}^{2}}{2\left(\tau_{i}^{2}+s_{i}^{2}\right)}}.$$ (18)

This is using the Gaussian density for *p_i_*. In most cases, the weights *w_i_* would be taken to be 1/*k* representing a view that the laboratories’ data are of equal quality. The mean of this distribution, that is, the average of the *k* laboratories measurements would be taken as the KCRV. The standard deviation of this distribution

$$u\left(\bar{y}\right)=\sqrt{\frac{\sum_{i}\left(\tau_{i}^{2}+s_{i}^{2}\right)}{k}+\frac{\sum_{i}{\left(y_{i}-\bar{y}\right)}^{2}}{k}}$$ (19)

being the standard uncertainty of the KCRV. The linear opinion pool is an easily understood and easily performed method. It is intuitively pleasing because the weights can be thought to represent the relative quality of the laboratories’ results. The estimator, with a frequentist interpretation, was used in Refs. [3] and [25]. Its main appeal in the Bayesian context, is that $u\left(\bar{y}\right)$ can be thought to represent the total variability in the population of measurands of the Key Comparison. This can be viewed as the true measure of uncertainty in such a Key Comparison, because of the assumed equality of the laboratories in terms of their competence.

## 6. Example: Analysis of CCAUV.V-K1

The Key Comparison in Vibration Acceleration is an interesting example of Class 1 Key Comparison with a traveling artifact. The data is given in Sect. 3.1. Table 1 summarizes the results of the analysis. Using Eq. (9), various results can be obtained depending on the value of *γ* ^2^. In Table 2, the results for *γ* = 0 are labeled “common mean model.” The results using a uniform prior distribution on *γ* are labeled “lab-effect model.” The third column contains the *Supra Bayes* estimate, one with *c_i_* = 1.0 and *a_i_* = 2.0, that is, 0.5 < *κ_i_* < 2.0, (*i* = 1,…,12). This choice of *c_i_* and *a_i_* gives a reasonable range of possible values for the standard uncertainties in this particular experiment as the declared standard uncertainties based on Table 1 range from a minimum of about one third of the average standard uncertainty to a maximum of about twice the average. The fourth column contains the results based on the *linear pool* estimator. In this particular Key Comparison, the separate measurands model is possibly too extreme as there truly was a single measurand. However, the separate measurands model can be thought of as a limiting case of a systematic lab effects model and as such can be used to obtain an upper bound on the uncertainty of the KCRV.

It is clear from Table 2 that even though the values of the Bayesian KCRV estimates under the various assumptions are very similar, their uncertainties are not. Thus, it is clearly important to examine the assumptions underlying the various analyses and make sure that they are reasonable. Equation (9), without the restriction on *γ*, quite objectively estimates the underlying variability due to systematic lab effects. It allows for the uncertainty to be as low as that given in Eq.(11), when the data supports it. In this case, the data clearly indicates that the uncertainty is larger. The posterior mean of *γ* is 1.9421E-4 with posterior standard deviation of 1.5838E-4. This gives a 95 % Highest Posterior Density Interval (HPD) of (8.2604E-6, 5.9033E-4). This is the shortest interval of possible values for *γ* which has probability of 0.95. For comparison, the individual laboratories’ Type B evaluated uncertainties ranged from 1.8974E-4 to 1.3153E-3. Note that these included terms based on random laboratory effects (estimated by *γ*) as well as terms based on other factors. Thus on the whole, the stated Type B evaluated uncertainties are reasonable, and the lab-effects-model KCRV would make a good choice.

The published analysis of CCAUV.V-K1 used Eqs. (10) and (11) for the KCRV and its uncertainty. The analysis (referred to as the common mean model above) was described as “correctly reflecting the declared uncertainties of the individual laboratories.” There is a discussion in the report, from a frequentist perspective, of statistical issues concerning the underlying assumptions of this analysis. Because of concerns with the validity of these assumptions, other frequentist estimates of the KCRV, including the average $\bar{y}$, the median of the *y_i_*, and a Maximum Likelihood Estimator (Ref. [23]) were computed. The report concluded that the values of these estimators were similar enough to justify the use of the common mean model for the KCRV in this key comparison. The report did not explicitly show the uncertainties associated with the various estimators.

## 7. Conclusions

Key Comparison experiments, performed in various sub-disciplines of physics and chemistry, pose numerous challenges to the analyst. Most importantly, Type B evaluated uncertainty must be included in the statistical model in a meaningful way, one that satisfies both the scientist and the statistician. Further, the scientific objectives of the experiment must be reflected in the statistical summaries and the results must be compliant with the *Guide to the Expression of Uncertainty in Measurement.* It is shown in this paper, that the Bayesian paradigm allows flexible modeling of Type B evaluated uncertainty and that it can produce estimates that are satisfactory to the needs of the scientists.

## Figures and Tables

**Table 1:** Charge sensitivity and the associated uncertainties

Laboratory number	*y_i_* in pC/(m/s^2^)	*s_i_* in 10^5^ pC/(m/s^2^)	*τ_i_* in 10^5^ pC/(m/s^2^)
1	0.12901	3.6	19
2	0.12914	5.5	73
3	0.12924	8.9	57
4	0.12874	6.1	66
5	0.12960	55.9	85
6	0.12890	3.9	72
7	0.12875	2.4	43
8	0.12870	9.6	39
9	0.12853	9.9	53
10	0.12830	4.4	54
11	0.12950	5.5	43
12	0.12877	11.6	132

**Table 2:** Comparison of KCRV estimates

	Common mean model pC/(m/s^2^)	Lab-effects model pC/(m/s^2^)	Supra-Bayes pC/(m/s^2^)	Linear-Pool pC/(m/s^2^)
KCRV	0.12892	0.12894	0.12894	0.12893
Uncertainty	0.000128	0.000167	0.000253	0.000791

## References

[1] (1999). Mutual Recognition of national measurements standards and of calibration and measurement certificates issued by national metrology institutes.

[2] CIPM (1999). Guidelines for CIPM key comparisons.

[3] Ciarlini P, Cox M, Pavese F, Regoliosi G (2004). The Use of a Mixture of Probability Distributions in Temperature Interlaboratory Comparisons. Metrologia.

[4] Pavese F (2004). Compound Modelling of Metrological Data Series, Advanced Mathematical and Computational Tools in Metrology.

[5] ISO Technical Advisory Group (1993). Working Group 3, Guide to the Expression of Uncertainty in Measurement.

[6] Cox MG (2002). The Evaluation of Key Comparison Data. Metrologia.

[7] Elster C, Link A (2000). Analysis of key comparison data: Assessment of current methods for determining a reference value. Measurement Science and Technology.

[8] Sutton C (2004). Analysis and linking of international measurement comparisons. Metrologia.

[9] White D (2004). On the analysis of measurement comparisons. Metrologia.

[10] Zhang NF, Liu HK, Sedransk N, Strawderman W (2004). Statistical analysis of key comparisons with linear trends. Metrologia.

[11] Kacker R, Jones A (2003). On use of Bayesian statistics to make the Guide to the Expression of Uncertainty in Measurement consistent. Metrologia.

[12] Wang C, Iyer H (2005). Propagation of uncertainties in measurements using generalized inference. Metrologia.

[13] Von Martens H, Elster C, Link A, Taubner A, Wabinski W, Will H (2001). Report on Key Comparison CCAUV.V-K1, PTB-1.22.

[14] ISO (1999). Methods for the calibration of vibration and shock transducers—Part 11, Reference number ISO 16063-11:1999(E).

[15] Toman B (2006). Linear statistical models in the presence of systematic effects requiring a Type B evaluation of uncertainty. Metrologia.

[16] Lindley DM, Smith AFM (1972). Bayes estimates for the linear model. J Roy Statist Soc B.

[17] Kacker R, Datla R, Parr A (2004). Statistical analysis of CIPM key comparisons based on the ISO Guide. Metrologia.

[18] Genest C, Zidek JV (1986). Combining probability Distributions: A Critique and Annotated Bibliography. Statistical Sci.

[19] Clemen RT, Winkler RL (1997). Combining Probability Distributions from Experts in Risk Analysis. Risk Analysis.

[20] Lindley DM (1983). Reconciliation of Probability Distributions. Operations Res.

[21] Jeffreys H (1963). Theory of Probability.

[22] Box G, Tiao G (1973). Bayesian Inference in Statistical Analysis.

[23] Vangel M, Rukhin A (1998). Estimation of a Common Mean and Weighted Means Statistics, J. Amer. Statistical Assoc.

[24] D’Agostini G (1999). Skeptical Combination of Experimental Results: General Considerations and Application to *ε′/ε*. CERN-EP/99–139.

[25] Duewer DL (2004). A Robust Approach for the Determination of CCQM Key Comparison Reference Values and Uncertanties. Working Document CCQM 04-15, BIPM.

